# A Pilot Randomized Controlled Trial of a Digital Intervention Aimed at Improving Food Purchasing Behavior: The Front-of-Pack Food Labels Impact on Consumer Choice Study

**DOI:** 10.2196/formative.9910

**Published:** 2019-04-08

**Authors:** Richard A Harrington, Peter Scarborough, Charo Hodgkins, Monique M Raats, Gill Cowburn, Moira Dean, Aiden Doherty, Charlie Foster, Edmund Juszczak, Cliona Ni Mhurchu, Naomi Winstone, Richard Shepherd, Lada Timotijevic, Mike Rayner

**Affiliations:** 1 Centre on Population Approaches for Non-Communicable Disease Prevention Nuffield Department of Population Health University of Oxford Oxford United Kingdom; 2 Food, Consumer Behaviour and Health Research Centre Faculty of Health & Medical Sciences University of Surrey Guildford United Kingdom; 3 Institute for Global Food Security School of Biological Sciences Queen's University Belfast Belfast United Kingdom; 4 Big Data Institute Nuffield Department of Population Health University of Oxford Oxford United Kingdom; 5 Centre for Exercise, Nutrition and Health Sciences School for Policy Studies University of Bristol Bristol United Kingdom; 6 National Perinatal Epidemiology Unit Nuffield Department of Population Health University of Oxford Oxford United Kingdom; 7 National Institute for Health Innovation School of Population Health University of Auckland Auckland New Zealand; 8 Department of Higher Education University of Surrey Guildford United Kingdom

**Keywords:** diet, randomized controlled trial

## Abstract

**Background:**

Most food in the United Kingdom is purchased in supermarkets, and many of these purchases are routinely tracked through supermarket loyalty card data. Using such data may be an effective way to develop remote public health interventions and to measure objectively their effectiveness at changing food purchasing behavior.

**Objective:**

The Front-of-pack food Labels: Impact on Consumer Choice (FLICC) study is a pilot randomized controlled trial of a digital behavior change intervention. This pilot trial aimed to collect data on recruitment and retention rates and to provide estimates of effect sizes for the primary outcome (healthiness of ready meals and pizzas purchased) to inform a larger trial.

**Methods:**

The intervention consisted of a website where participants could access tailored feedback on previous purchases of ready meals and pizzas, set goals for behavior change, and model and practice the recommended healthy shopping behavior using traffic light labels. The control consisted of Web-based information on traffic light labeling. Participants were recruited via email from a list of loyalty card holders held by the participating supermarket. All food and drink purchases for the participants for the 6 months before recruitment, during the 6-week intervention period, and during a 12-week washout period were transferred to the research team by the participating supermarket. Healthiness of ready meals and pizzas was measured using a predeveloped scale based solely on the traffic light colors on the foods. Questionnaires were completed at recruitment, end of the intervention, and end of washout to estimate the effect of the intervention on variables that mediate behavior change (eg, belief and intention formation).

**Results:**

We recruited 496 participants from an initial email to 50,000 people. Only 3 people withdrew from the study, and purchase data were received for all other participants. A total of 208 participants completed all 3 questionnaires. There was no difference in the healthiness of purchased ready meals and pizzas between the intervention and control arms either during the intervention period (*P*=.32) or at washout (*P*=.59).

**Conclusions:**

Although the FLICC study did not find evidence of an impact of the intervention on food purchasing behavior, the unique methods used in this pilot trial are informative for future studies that plan to use supermarket loyalty card data in collaboration with supermarket partners. The experience of the trial showcases the possibilities and challenges associated with the use of loyalty card data in public health research.

**Trial Registration:**

ISRCTN Registry ISRCTN19316955; http://www.isrctn.com/ISRCTN19316955 (Archived by WebCite at http://www.webcitation.org/76IVZ9WjK)

**International Registered Report Identifier (IRRID):**

RR2-10.1186/s40814-015-0015-1

## Introduction

### Background

Poor diet is a major risk factor for noncommunicable diseases (NCDs) in the United Kingdom, responsible for more than 10% of all morbidity and mortality [1]. Food purchasing precedes and affects food consumption, which makes food purchasing environments a prime setting for intervention studies aimed at improving diet and nutrition. In the United Kingdom, most food shopping is conducted in supermarkets [2], and supermarket purchases have been shown to correlate well with food and nutrient consumption [3].

**Figure 1:**
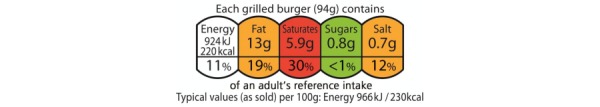
Example of the type of front of pack labeling recommended by the UK Government for a packet of 4 beef burgers, containing numeric information, percentage of reference intakes, and traffic light color coding.

Front of pack (FOP) nutrition labeling on food packaging has been used in the United Kingdom since the mid-2000s [4]. In October 2012, traffic light labeling of nutrients was recommended by the UK Government for FOP labeling [5], and it is currently being used by many UK manufacturers and retailers. Traffic light labeling involves a color-coded assessment (green for low, amber for medium, and red for high) of the level of total fat, saturated fat, sugar, and salt (see Figure 1 [6]).

Supermarket loyalty card data are a potential source of “big data” that could allow for remote, objective monitoring of food purchasing behavior, enabling interventions aimed at improving the healthiness of food purchases that could be delivered at scale to a large population as they require no additional burden beyond continued use of loyalty cards during routine food purchasing. However, loyalty card data are owned by the supermarket industry, and little is known about the feasibility of using such data for the development of public health interventions that incorporate tailored feedback on previous purchases. This is because loyalty card data are commercially sensitive and consumers have privacy concerns over the handling of loyalty card data [7]; as such, it is typically difficult to access. Therefore, the use of such data is rare in the evaluation of public health interventions [8-11].

This study reports on a pilot 2-arm equal allocation parallel randomized controlled trial (RCT) of a digital intervention that incorporated a number of behavior change techniques. The intervention consists of a password-protected website where users can access tailored feedback on previous purchases of ready meals and pizzas, set goals for behavior change, and model and practice the recommended healthy shopping behavior. A theoretical approach based on selection of the most relevant behavior change mechanisms was adopted rather than utilization of an entire theoretical framework, as it has been suggested that this is an optimal approach in a food context [12]. The intervention was aimed at increasing the use of traffic light labeling to encourage healthier purchase decisions when purchasing ready meals and pizzas, thereby leading to purchases lower in total fat, saturated fat, sugar, and salt. Design of the intervention was based on reviews of behavior change literature, and previous research has shown that remotely delivered interventions that provide tailored feedback on previous behavior can improve dietary behavior [13].

The pilot trial was part of the Front-of-pack food Labels: Impact on Consumer Choice (FLICC) project. The protocol for the pilot trial has been previously published and includes a detailed description of development of the intervention design and content [14], and the pilot trial was registered at the ISRCTN registry (ISRCTN19316955).

### Objectives

The objectives of the trial were to assess the feasibility of a full RCT by measuring recruitment, retention, and data completion rates of participants, producing estimates for the potential effect size of the intervention on healthiness of purchased own-brand ready meals and pizzas—the primary outcome measure, and producing estimates for the potential effect size of the intervention on all food purchases, purchases of fruit and vegetables, and psychosocial variables associated with label use—secondary outcome measures.

Our hypotheses were that the intervention would increase the healthiness of purchased ready meals and pizzas, while not affecting the total amount of ready meals and pizzas purchased, nor affecting purchasing behavior in other food categories. We hypothesized that the intervention would operate by impacting on mechanisms affecting beliefs and behavioral intention formation as well as those associated with planning and goal setting and the adoption and maintenance of the behavior of interest, namely, traffic light labeling use during purchases of ready meals and pizzas. Due to constraints in the availability of data and the fact that not all branded products contain traffic light labels, we limited our analyses to supermarket’s own-brand products only. We hypothesized that the majority of ready meal and pizza purchases would be own-brand products, and so, restricting analyses to these product lines would have limited effect on results.

## Methods

**Figure 2:**
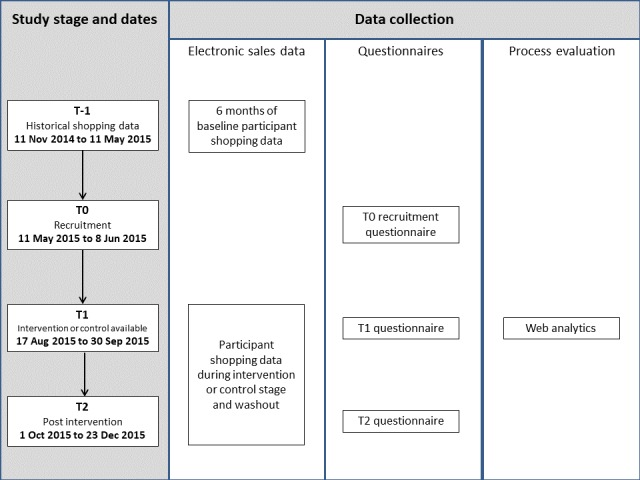
Outline of trial calendar, illustrating data collected at each stage.

Data collection for the FLICC pilot trial took place over 58 weeks from November 11, 2014, to December 23, 2015. The data collected comprised food purchase data obtained from the supermarket loyalty card database and self-completion participant questionnaire data. The trial was split into 4 distinct time periods: 26 weeks of baseline historical shopping data (T-1), 4 weeks of recruitment (T0), 6 weeks of intervention (T1), and 12 weeks of follow-up without intervention (T2). A further 10 weeks between the end of T0 and the start of T1 were used to request, receive, and process the shopping history data for use in the intervention. The trial stages and types of data collected at each point are shown in Figure 2.

The primary focus of this trial was purchases of own-brand ready meals and pizzas. These food categories were chosen because they are highly likely to carry traffic light labeling in the participating supermarket; there is considerable nutritional variation in these food categories, allowing participants scope for buying healthier products; and ready meals and pizzas represent a large and growing proportion of food sales in the United Kingdom [14,15].

### Ethical Approval

Ethics was granted by the University of Oxford Central University Research Ethics Committee (SSD/CUREC1/14-008) and the University of Surrey Ethics Committee (EC/2014/153/FAHS).

### Recruitment

To be eligible for the FLICC pilot trial, individuals needed to be UK residents, have had a loyalty card with the participating supermarket for at least 6 months at recruitment, be older than 18 years, do most of their shopping at stores larger than 8000 square feet (this criterion was to ensure that participants would have access to a large supply of own-brand ready meals and pizzas), be the primary food shopper for their household, not be planning to leave the United Kingdom for longer than 3 weeks during the study period, and have purchased at least 10 ready meals and pizzas in the previous 6 months (self-reported).

On the basis of a power calculation shown in the protocol [14], we aimed to recruit 1300 participants by email from a loyalty card database of the participating supermarket, using a passive approach—a recruitment method that requires a potential participant to make the first contact with the study following an invitation [16]. The supermarket chain emailed 50,000 cardholders selected at random in batches of 3000 from the whole population of cardholders, after filtering to exclude individuals who would not meet the first 4 inclusion criteria. The recruitment email included information on the trial and a link to a FLICC registration website, where participants were screened for the remaining 3 eligibility criteria, provided extra information for allocation through block randomization, and gave informed consent for participation in the trial.

### Allocation

Block randomization was used to allocate individuals to the intervention or control arm, stratifying by sex and whether or not participants had dependent children. Participants were told the study was about the influence of traffic light labels on purchasing decisions but not informed whether they were in the intervention or control arm. A total of 2 researchers (RAH and PS) implemented the randomization and had access to the list of control and intervention participants during the study.

### Intervention

A full description of the intervention is provided in the published trial protocol [14]; however, a summary of the key components is given in Table 1. A theoretical approach based on the selection of the most relevant behavior change mechanisms was adopted [12,17], and the intervention contains both passive components (information delivery via the Web application) and interactive components (participant is encouraged to engage with the Web application). The passive and interactive elements of the intervention are highlighted in Table 1. Overall, the intervention was designed to help people make intracategory decisions (eg, to compare pizza A and pizza B) and by focusing only on the use of the traffic light element (ie, colors) of the nutrition label.

Participants in each arm were sent an email containing a URL to a password-protected Web application, which remained open for 6 weeks (T1). The control group received a subset of the digital intervention: information on the importance of healthy eating, a description of traffic light labeling, and the message “Green is better than amber but amber is better than red!” Screenshots of the intervention are available from the Centre on Population Approaches for NCD Prevention website [18] or by request to the corresponding author.

### Data Collection

Data on food purchases by the participants were collected by the participating supermarket’s loyalty card system. Data on all foods and drinks purchased (while using the loyalty card) by the participants in any store across the United Kingdom during the T-1 period were transferred by the participating supermarket to the research team after recruitment had closed. A second transfer of equivalent data covering the periods T1 and T2 was conducted after the study had finished. Where participants withdrew from the study, food purchase data were transferred only up to the withdrawal date, and their questionnaire data were not included for secondary outcome analyses.

The participating supermarket also provided data on the nutritional quality of all own-brand food products currently on sale at 2 time points (before recruitment and after the study had completed), which were used to derive traffic light labels and to calculate outcome measures. Nutrition data for own-brand products found in the shopping history data that were not present in the supermarket nutrition dataset were extracted from the Brandbank database [19].

Demographic (age, sex, ethnicity, educational status, and household size) and socioeconomic (income and job classification) data were collected in the first of 3 Web-based questionnaires delivered at recruitment (T0). Psychosocial variables were collected at T0 and at the end of T1 and T2. In addition, Web analytics were collected to provide data on participants’ engagement with the intervention. A single reminder was sent out for completion of the second and third questionnaires, which were incentivized by a £10 online gift voucher.

### Measures

This pilot trial collected data on recruitment and retention rates and estimates of effect size for the primary and secondary outcome variables. The primary outcome measure for this trial was the healthiness of own-brand ready meals and pizzas that had traffic light labeling measured at both T1 and T2, where “healthiness” of each item was calculated from a combination of the information provided on the traffic light label. The score ranges from 0 (for 4 red lights) to 1 (for 4 green lights). Foods are awarded 0.15 points for each amber light and 0.25 points for each green light. The weighting for the different colors was derived from a choice experiment [20] where participants were asked to decide between the healthiness of 2 foods based purely on the traffic light label information, thereby revealing the different prominence awarded to each color in the decision-making process.

**Table 1:** Intervention components.

Behavior change techniques	Intervention components	Behavioral mechanisms impacted
Provide information on consequences of behavior to the individual	The risks of eating a diet high in fat, saturated fat, salt, and sugar and the prominence of these nutrients in ready meals and pizzas are reported (passive)^a^. Personalized feedback on the traffic light profile of the 6 months of ready meals and pizzas purchased by the participant in T1 study period are delivered. Participants are presented with an infographic summarizing the 6 months of data and are able to interrogate the previous data in simple tables, with comparisons made with other available products (interactive).	Mechanisms affecting belief formation and cognitive mechanisms: attention bias, optimistic bias
Provide instruction (how to perform the behavior)	A description is provided of the traffic light labeling that the participants will find in the participating supermarket and what the traffic light colors mean (passive)^a^.	Mechanisms of intention formation: outcome expectancies, (action) self-efficacy, and perceived behavioral control; heuristics
Provide information about the traffic light label	Information about the traffic light label profile of a selection of the ready meals and pizzas that are available from the participating supermarket is provided in a tabular form that the participant can interrogate. Designed to highlight the potential for nutritional improvement within the ready meals and pizzas categories (interactive).	
Goal setting	The following outcome goal is provided: “Use traffic light labels when you are shopping in (participating supermarket) for ready meals and pizzas. Compare the traffic light labels between products and try to buy healthier ready meals and pizzas than you would normally. You can do this by reducing the number of red lights on the label and increasing the number of green lights on the label” (passive).	Planning and goal setting
Modeling the behavior	A short video showing individuals performing the behavior in a real store will be provided (passive).	Mechanisms of intention formation: Outcome expectancies and (Action) self-efficacy; perceived behavioral control
Prompt practice	An experiential task is provided, which allows participants to increase their self-efficacy in using traffic light food labels. This consists of multiple-choice tests asking participants to choose healthier versions of ready meals or pizzas with and without traffic light information provided. The intention is to demonstrate that the traffic light information can make these decisions easier to make (interactive).	Mechanisms of intention formation: (Action) self-efficacy; perceived behavioral control
Action planning	Participant is encouraged to plan when and where they will perform the desired behavior via the development of intention statements which they then enter into the Web application (interactive).	Planning and goal setting

^a^This element is provided to participants in both the intervention and the control arm.

In a sample of 406 ready meals and pizzas from the participating supermarket collected in November 2013, the healthiness score was reasonably normally distributed with a mean of 0.63 and SD of 0.21. Foods with scores of 0 and 1 were identified in the sample, demonstrating that the full range of the score was used.

The secondary outcome measures were as follows:

1. The number of ready meals and pizzas purchased in T1/T2
2. Expenditure (measured in £) on ready meals and pizzas purchased in T1/T2 
3. The total amount (measured in grams) of fat, saturated fat, sugar, and salt in ready meals and pizzas purchased in T1/T2
4. Expenditure (measured in £) on all foods purchased in T1/T2 
5. Expenditure (measured in £) on fruit and vegetables purchased in T1/T2
6. Psychosocial variables including “Stage model of health awareness” [21,22], “Perceived intake,” “Perceived need to change,” “Expectation and Intention” [23], and “Potential barriers to labelling use” [24-26] measured in T1/T2.

These psychosocial variables were selected to measure the effectiveness of the mechanisms we employed in the intervention design (Table 2) in terms of their potential to shift participants to using traffic light labeling (stage model of health awareness) by impacting on belief formation (perceived intake and perceived need to change), intention formation, and expectation. Potential barriers to labeling use were also measured.

**Table 2:** Psychosocial variables questions and response options.

Variable	Intervention text	Response
Stage model of health awareness adapted from Weinstein & Sandman and Renner & Schwarzer [21,22]	Thinking about the color-coded nutrition labels often referred to as “traffic light labels,” which can be found on the front of food packaging, please select 1 of the following statements which most applies to you.	(1) I have never thought about using Traffic Light Labels when I shop. (2) I have thought about using Traffic Light Labels when I shop but I don’t need to do anything. (3) I have thought about using Traffic Light Labels when I shop but I am still undecided. (4) I have already planned to use Traffic Light Labels when I shop but I haven’t done anything yet. (5) I am using Traffic Light Labels when I shop and intend to continue doing so in future.
Perceived intake adapted from Raats et al [23]	Thinking about the number of reds on the traffic light labels of the ready meals/pizzas that you typically purchase, how low or high do you think this is?	(1) Extremely low-(7) extremely high
Perceived need to change adapted from Raats et al [23]	To what extent do you feel that you need to use traffic light labels over the next 6 weeks to help you choose ready meals/pizzas that are healthier?	(1) Definitely do not need to-(7) definitely need to
Expectation adapted from Raats et al [23]	How likely/unlikely is it that you will use traffic light labels over the next 6 weeks to help you choose ready meals/pizzas that are healthier?	(1) Extremely unlikely-(7) extremely likely
Intention adapted from Raats et al [23]	I intend to use traffic light labels over the next 6 weeks to help me choose healthier ready meals/pizzas?	(1) Definitely do not-(7) definitely do
Potential barriers to labeling use informed by Cowburn, Cowburn & Stockley and Grunert & Wills [24-26]	In my opinion traffic light labelling...is confusing to use; is truthful; is accurate; is hard to understand; is interesting to use; means you have to do math; means you need to know a lot about nutrition.	(1) Strongly disagree-(7) strongly agree

### Statistical Analysis

All analyses were conducted in accordance with a predetermined statistical analysis plan, available from the Centre on Population Approaches for NCD Prevention website [18] or by request to the corresponding author. For normally distributed outcomes, analysis of covariance was used to assess differences between intervention and control arms at periods T1 and T2, adjusted for sex, whether or not the participants had dependent children, and measures collected in T-1 using the following model proposed by Vickers and Altman [27], illustrated by the primary outcome analysis at T1, where beta are regression coefficients, ε is an error term, and “group” refers to allocation to intervention or control:

Healthiness
_T1_ = β
_0_ + β
_1_*Sex + β
_2_*Dependents + β
_3_*Healthiness
_T-1_ + β
_4_*Group + ε

Where not normally distributed, differences were assessed using Mann-Whitney *U* tests not adjusted for sex, dependent children, or measures collected at T-1. Predetermined subgroup analyses of the primary outcome measure were conducted, stratified by socioeconomic status based on job classification (National Statistics Socio-economic Classification (NS-SEC) 1 or 2 vs 3 to 5) using a standard UK definition [28].

Within this study, missing outcome data (MOD) occurred for a number of reasons. For both the sales data and questionnaire data, MOD were generated by participants withdrawing post randomization. For the questionnaire data, MOD were generated by failure to complete some or all of the questions within a questionnaire. The primary outcome variable (average healthiness of ready meals and pizzas purchased in T-1, T1, and T2) included MOD if the participant did not purchase any own-brand ready meals or pizzas using their loyalty card in any of the 3 study phases. A systematic review of methods used to cope with MOD in intention-to-treat analyses demonstrated that there is no consensus toward a preferred approach, with arguments for restricting to complete case analysis and for imputation of missing data [29]. For the FLICC study, we dealt with MOD in the sales data by employing imputation techniques (multiple imputation for the primary outcome variable and single imputation for the secondary outcome variables). The imputation method used regression analysis with sex and dependent children as predictor variables as these were used in the block allocation, so there was no chance of missing data. The imputation datasets were all observations within T-1 for MOD at T-1 and equivalent for periods T1 and T2. We assessed the nature of the MOD from the primary outcome variable by conducting chi-square tests of the presence of missing data at any of the 3 study periods with sex and dependent children. For the questionnaire data, where retention rates were expected to be lower, we conducted analysis on a complete case basis (defined as being participants that provided electronic sales data and questionnaire data at all time points) as predetermined by our statistical analysis plan.

## Results

### Results to Predetermined Analyses

Of the 50,000 loyalty cardholders who received invitation emails, 869 clicked the link to the FLICC recruitment website. Of these, 496 were eligible and completed the consent process. These figures are illustrated in the flowchart in Multimedia Appendix 1. Our passive recruitment method recruited approximately 1% (496/50000) of the participant pool.

**Table 3:** Recruitment, retention, and data completeness by sex, dependents, socioeconomic status, ethnicity, age, educational status, general health interest, and dietary considerations because of health status.

Characteristic			Intervention group (n=246), n (%)	Control group (n=250), n (%)	Participants with complete^a^ data (n=208), n (%)	Participants with incomplete data (n=288), n (%)	*P* value^b^
**Sex**							
	Male		82 (33.3)	81 (32.4)	70 (33.7)	93 (32.3)	.89
	Female		164 (66.7)	169 (67.6)	138 (66.3)	195 (67.7)	.89
**Dependent children**							
	Yes		80 (32.5)	80 (32.0)	62 (29.8)	98 (34.0)	.33
	No		166 (67.5)	170 (68.0)	146 (70.2)	190 (66.0)	.33
**Socioeconomic status**							
	Managerial and professional occupations		140 (56.9)	135 (54.0)	149 (71.6)	126 (43.8)	.40
	Intermediate occupations		15 (6.1)	10 (4.0)	10 (4.8)	15 (5.2)	.40
	Small employers and own account workers		14 (5.7)	19 (7.6)	16 (7.7)	17 (5.9)	.40
	Lower supervisory and technical operations		9 (3.7)	9 (3.6)	7 (3.4)	11 (3.8)	.40
	Semiroutine and routine occupations		20 (8.1)	21 (8.4)	24 (11.5)	17 (5.9)	.40
	Undisclosed or missing data		48 (19.5)	56 (22.4)	2 (1.0)	102 (35.4)	.40
**Ethnicity**							
	White		192 (78.0)	188 (75.2)	198 (95.2)	182 (63.2)	.62
	Mixed/multiple ethnic groups		1 (0.4)	0 (0)	1 (0.5)	0 (0)	.62
	Asian/Asian British		0 (0)	3 (1.2)	2 (1.0)	1 (0.3)	.62
	Black/African/Caribbean/Black British		1 (0.4)	2 (0.8)	1 (0.5)	2 (0.7)	.62
	Other ethnic group		1 (0.4)	0 (0)	1 (0.5)	0 (0)	.62
	Undisclosed or missing data		51 (20.7)	57 (22.8)	5 (2.4)	103 (35.8)	.62
**Age (years)**							
	18-25		5 (2.0)	5 (2.0)	4 (1.9)	6 (2.1%)	.78
	26-35		24 (9.7)	16 (6.4)	26 (12.5)	14 (4.9)	.78
	36-45		41 (16.7)	45 (18.0)	45 (21.6)	41 (14.2)	.78
	46-55		62 (25.2)	50 (20.0)	59 (28.4)	53 (18.4)	.78
	56-65		37 (15.0)	54 (21.6)	48 (23.1)	43 (14.9)	.78
	66-75		24 (9.7)	21 (8.4)	21 (10.1)	24 (8.3)	.78
	76+		5 (2.0)	4 (1.6)	4 (1.9)	5 (1.7)	.78
	Undisclosed or missing data		48 (19.5)	55 (22.0)	1 (0.5)	102 (35.4)	.78
**Educational status^c^**							
	(1)		6 (2.4)	4 (1.6)	6 (2.9)	4 (1.4)	.51^f^
	(2)		14 (5.7)	6 (2.4)	8 (3.8)	12 (4.2)	.51^f^
	(3)		27 (11.0)	25 (10.0)	25 (12.0)	27 (9.4)	.51^f^
	(4)		0 (0)	1 (0.4)	0 (0)	1 (0.3)	.51^f^
	(5)		38 (15.5)	38 (15.2)	46 (22.1)	30 (10.4)	.51^f^
	(6)		65 (26.4)	74 (29.6)	74 (35.6)	65 (22.6)	.51^f^
	(7)		41 (16.7)	46 (18.4)	45 (21.6)	42 (14.6)	.51^f^
	(8)		6 (2.4)	2 (0.8)	2 (1.0)	6 (2.1)	.51^f^
	(9) Undisclosed or missing		49 (19.9)	54 (21.6)	2 (1.0)	101 (35.1)	.51^f^
**General health interest^d^**							
	Low health interest		17 (6.9)	18 (7.2)	20 (9.6)	15 (5.2)	.42
	High health interest		189 (76.8)	187 (74.8)	188 (90.4)	188 (65.3)	.42
	Missing data		40 (16.3)	45 (18.0)	0 (0)	85 (29.5)	.42
**Dietary considerations because of health status^e^**							
	**Coronary heart disease/high blood pressure**						
		Yes	64 (26.0	72 (28.8)	66 (31.7)	70 (24.3)	.55
		No	142 (57.7)	133 (53.2)	142 (68.3)	133 (46.2)	.55
		Missing data	40 (16.3)	45 (18.0)	0 (0)	85 (29.5)	.55
	**Weight management/obesity**						
		Yes	96 (39.0)	105 (42.0)	101 (48.6)	100 (34.7)	.89
		No	110 (44.7)	100 (40.0)	107 (51.4)	103 (35.8)	.89
		Missing data	40 (16.3)	45 (18.0)	0 (0)	85 (29.5)	.89
	**Type 2 diabetes**						
		Yes	27 (10.9)	30 (12.0)	30 (14.4)	27 (9.4)	.74
		No	179 (72.8)	175 (70.0)	178 (85.6)	176 (61.1)	.74
		Missing data	40 (16.3)	45 (18)	0 (0)	85 (29.5)	.74

^a^“Participants with complete data” refers to all participants for which a complete set of electronic sales data and questionnaire data at 3 time points is available. Some missing data still arise from within the questionnaire data where participants chose not to respond to a particular question. For all variables, the difference was assessed excluding missing data.

^b^Difference between complete versus incomplete data participants. Difference is assessed with Pearson chi-square test excluding missing data.

^c^(1) No qualifications; (2) 1-4 O levels /certificate of secondary education (CSE)/general certificate of secondary education (GCSEs; any grades), entry level, foundation diploma, national vocational qualification (NVQ) level 1, foundation general national vocational qualification (GNVQ), basic/essential skills; (3) 5 or more O level (passes)/CSEs (grade 1)/GCSEs (grades A*-C), school certificate, 1 A level/2 to 3 advanced subsidiary levels/Victorian Certificate of Education (VCEs), intermediate/higher diploma, intermediate diploma, NVQ level 2, intermediate GNVQ, City and Guilds Craft, BTEC first/general diploma, Royal Society of Arts (RSA) diploma; (4) apprenticeship; (5) 2 or more A levels/VCEs, 4 or more AS Levels, higher school certificate, progression/advanced diploma, NVQ Level 3—advanced GNVQ, City and Guilds Advanced Craft, ONC, OND, BTEC, National, RSA advanced diploma; (6) degree (eg, BA and BSc), higher degree (eg, MA, PhD, and PGCE), NVQ Level 4-5, HNC, HND, RSA higher diploma, BTEC higher level, foundation degree; (7) professional qualifications (eg, teaching, nursing, and accountancy); (8) other: vocational/work-related qualifications, qualifications gained outside the United Kingdom; and (9) undisclosed or missing.

^d^General health interest [30].

^e^Dietary considerations due to health status: “When buying food for yourself or your family do you have to consider dietary requirements relating to any of the following? Coronary Heart disease/High blood pressure; Weight management/Obesity; Type 2 Diabetes.” Response options Yes/No.

^f^Chi-square test performed on combined groups to avoid low numbers in cells.

Of the recruited participants, 3 withdrew without reason after randomization but before the T1 period commenced—complete purchase data were collected for all other participants. The completion rates for the recruitment (T0), second (T1), and third (T2) questionnaires were 79% (394/496), 54% (270/496), and 63% (313/496), respectively. A chi-square test showed no evidence of difference in provision of complete data by allocation group. Of the 496 recruited participants, 208 (42%) provided complete data (ie, completed all 3 questionnaires and allowed transferal of purchasing data for the complete study period). The majority of the participants were older than 46 years, white, female, with no dependent children, and were in high socioeconomic groups (NS-SEC 1 or 2; Table 3). More than three-quarters of the sample reported a high interest in health using a predefined measure [30].

During the intervention period, 438 ready meals and pizzas were available to purchase through the participating supermarket. A total of 317 (72.4%) of the products were supermarket own-brand and 121 (27.6%) were branded products. Of 10,416 ready meal and pizza purchases used in the analyses, 8263 (79.3%) were supermarket own-brand and 2153 (20.7%) were branded products.

In all 3 data collection periods, there were high levels of MOD for both control and intervention groups for the primary outcome (average healthiness of traffic light and ready meals), indicating zero recorded purchases of own brand ready meals and pizzas (Table 4). Assessments of the association between the presence of MOD and the 2-block randomization variables (sex and dependent children) showed no evidence of association (*P*>.2 in all cases), suggesting that the MOD were missing completely at random. The results for the primary outcome showed no difference between intervention and control groups during periods T1 (*P*=.32) and T2 (*P*=.59). Predetermined subgroup analyses stratified by socioeconomic status also did not find any differences between intervention and control groups (Multimedia Appendix 1). Exploratory analyses did not find a significant interaction between allocation group and socioeconomic status.

The difference between the control and intervention arms for the food purchasing secondary outcome measures are shown in Table 5 and reveal no evidence that the intervention changed purchasing behavior. In terms of the psychosocial secondary outcome measures (Table 6), these similarly demonstrate no difference between intervention and control groups for the effect of the intervention.

In the context of the lack of an observed effect in the primary outcome for this intervention, it is interesting to note that just over half the participants in the intervention arm (54%) logged onto the FLICC website (as measured by Web analytics) during the study period, and therefore, many participants did not receive or engage with the intervention material at all. Similar levels of engagement were observed in the control arms (48%). In terms of some of the key elements of the intervention content reported in Table 7, in all cases, less than half the intervention arm participants engaged with these.

### Post Hoc Results

A complete case analysis of the primary outcome variable, where participants are only included if they purchased at least one ready meal or pizza in both the baseline and either intervention period (n=213) or washout period (n=266), showed a significant increase in the healthiness of food purchases in the intervention group of 0.04 (*P*=.03)—roughly equivalent to switching 1 red light for 1 amber light for every 3 ready meals or pizzas purchased. There was no difference between intervention and control groups at washout in the complete case analysis.

**Table 4:** Primary outcome measure results—healthiness of ready meals and pizzas purchased by intervention and control arms in 3 study phases. Healthiness score range between 0 and 1, with a higher score indicating healthier food purchases (n=496).

Allocation group, followed by different definitions of missing data	Average healthiness of traffic lights for ready meals and pizzas^a^, T-1		Average healthiness of traffic lights for ready meals and pizzas^a^, T1		Average healthiness of traffic lights for ready meals and pizzas^a^, T2	
	Mean (SE)	*P* value	Mean (SE)	*P* value	Mean (SE)	*P* value
Control	0.561 (0.008)	.12	0.561 (0.009)	.32	0.557 (0.010)	.59
Intervention	0.582 (0.008)	.12	0.581 (0.010)	.32	0.555 (0.009)	.59
Missing data because of zero purchases of ready meals and pizza^b^, n (%)	111 (22.4)	—^c^	258 (52.0)	—	196 (39.5)	—
Missing data because of withdrawal^b^, n (%)	0 (0)	—	3 (0.6)	—	3 (0.6)	—

^a^Results of analysis of covariance comparing intervention and control adjusted for sex and dependent children at T-1 and sex, dependent children, and healthiness of ready meals and pizzas purchased at T-1 at other time points.

^b^Multiple imputation using stochastic regression with sex and dependent children as predictors was used to replace missing data in analyses.

^c^Not applicable.

**Table 5:** Secondary outcome measure results with purchase data using multiple imputation for missing data (3 cases for all variables because of participant withdrawal; n=496).

Variable			T-1				T1				T2		
			Mean (SE)		*P* value		Mean (SE)		*P* value		Mean (SE)		*P* value
**Number of ready meals/pizzas purchased (items per week)^a^**													
	Control	0.32 (0.03)		.81		0.32 (0.04)		.97		0.32 (0.04)		.57	
	Intervention	0.37 (0.04)		.81		0.34 (0.05)		.97		0.32 (0.03)		.57	
**Amount (£) of ready meals/pizzas purchased^a^**													
	Control	0.85 (0.09)		.99		0.84 (0.10)		.73		0.77 (0.09)		.52	
	Intervention	0.93 (0.10)		.99		0.88 (0.12)		.73		0.84 (0.09)		.52	
**Total fat (gram) purchased per week^a^**													
	Control	8.09 (0.71)		.81		7.98 (0.88)		.75		7.81 (0.89)		.51	
	Intervention	9.15 (0.84)		.81		7.83 (0.96)		.75		8.03 (0.79)		.51	
**Saturated fat (gram) purchased per week^a^**													
	Control	3.40 (0.31)		.91		3.37 (0.38)		.62		3.26 (0.40)		.49	
	Intervention	3.86 (0.37)		.91		3.18 (0.41)		.62		3.37 (0.35)		.49	
**Total sugar (gram) purchased per week^a^**													
	Control	3.27 (0.30)		.90		3.31 (0.37)		.82		3.33 (0.37)		.56	
	Intervention	3.56 (0.31)		.90		3.29 (0.42)		.82		3.19 (0.31)		.56	
**Salt (gram) purchased per week^a^**													
	Control	0.80 (0.07)		.91		0.77 (0.09)		.98		0.78 (0.09)		.55	
	Intervention	0.89 (0.09)		.91		0.80 (0.10)		.98		0.79 (0.08)		.55	
**Amount (£) of fruit and vegetables purchased^a^**													
	Control	2.16 (0.19)		.21		2.00 (0.20)		.21		1.64 (0.14)		.24	
	Intervention	2.09 (0.22)		.21		1.82 (0.20)		.21		1.64 (0.19)		.24	
**Amount (£) of all food purchased**													
	Control	18.99 (1.37)		.60		17.49 (1.34)		.43		17.45 (1.29)		.41	
	Intervention	19.03 (1.38)		.60		17.23 (1.37)		.43		16.57 (1.35)		.41	

^a^*P* values for difference between control and intervention adjusted for sex, dependent children, and results at T-1 using analysis of covariance or from unadjusted Mann-Whitney *U* test for non-normally distributed variables.

**Table 6:** Secondary outcome measure results for psychosocial variables for participants (n=208) with complete data (ie, all participants for which a complete set of electronic sales data and questionnaire data at 3 time points is available. Some missing data still arise from within the questionnaire data where participants chose not to respond to a particular question).

Psychosocial variable			T0		T1		T2	
			Mean (SE)	*P* value	Mean (SE)	*P* value	Mean (SE)	*P* value
**Stage model of health awareness [21,22]**								
	Control		3.62 (0.16)	.53	4.22 (0.13)	.14	4.47 (0.11)	.78
	Intervention		3.81 (0.14)	.53	4.39 (0.12)	.14	4.50 (0.10)	.78
**Perceived intake^a^** **[23]**								
	Control		4.92 (0.12)	.90	4.75 (0.13)	.99	4.82 (0.15)	.97
	Intervention		4.92 (0.10)	.90	4.74 (0.13)	.99	4.81 (0.14)	.97
**Perceived need to change [23]**								
	Control		5.27 (0.14)	.70	5.18 (0.16)	.47	5.25 (0.15)	.09
	Intervention		5.16 (0.15)	.70	5.05 (0.15)	.47	4.92 (0.15)	.09
**Expectation [23]**								
	Control		5.42 (0.14)	.44	5.14 (0.16)	.55	5.53 (0.16)	.36
	Intervention		5.30 (0.14)	.44	5.33 (0.13)	.55	5.41 (0.15)	.36
**Intention [23]**								
	Control		5.68 (0.15)	.05	5.17 (0.18)	.92	5.54 (0.15)	.32
	Intervention		5.33 (0.14)	.05	5.32 (0.15)	.92	5.35 (0.15)	.32
**Potential barriers to labeling use [24-26]**								
	**Confusing to use**							
		Control	2.33 (0.13)	.89	2.10 (0.12)	.78	1.98 (0.11)	.97
		Intervention	2.29 (0.10)	.89	2.07 (0.12)	.78	1.99 (0.11)	.97
	**Truthful**							
		Control	5.25 (0.12)	.21	5.32 (0.12)	.43	5.40 (0.11)	.33
		Intervention	5.03 (0.11)	.21	5.25 (0.11)	.43	5.24 (0.11)	.33
	**Accurate**							
		Control	5.01 (0.13)	.25	5.19 (0.13)	.42	5.34 (0.11)	.31
		Intervention	4.89 (0.10)	.25	5.14 (0.10)	.42	5.18 (0.11)	.31
	**Hard to understand**							
		Control	2.01 (0.12)	.08	2.02 (0.13)	.62	1.85 (0.10)	.41
		Intervention	2.20 (0.11)	.08	1.83 (0.09)	.62	1.96 (0.10)	.41
	**Interesting to use**							
		Control	5.10 (0.12)	.66	4.98 (0.14)	.79	5.03 (0.13)	.73
		Intervention	4.94 (0.13)	.66	5.01 (0.11)	.79	4.92 (0.14)	.73
	**Means you have to do maths**							
		Control	2.10 (0.13)	.89	2.16 (0.14)	.63	2.03 (0.12)	.66
		Intervention	2.07 (0.12)	.89	1.98 (0.11)	.63	2.10 (0.12)	.66
	**Means you need to know a lot about nutrition**							
		Control	2.62 (0.16)	.24	2.51 (0.15)	.22	2.26 (0.14)	.27
		Intervention	2.76 (0.14)	.24	2.18 (0.11)	.22	2.40 (0.13)	.27

^a^Differences assessed between control and intervention adjusted for sex and dependent children using repeated measures analysis of variance; all other differences are assessed by unadjusted Mann-Whitney U test for non-normally distributed variables.

**Table 7:** Participant engagement with the intervention measured by Web analytics (excluding withdrawn participants).

Activity	Randomized sample (n=493), n (%)	Control arm (n=248), n (%)	Intervention arm (n=245), n (%)
Logged onto FLICC^a^ website	251 (50.6)	120 (48.4)	131 (53.5)
Watched video	—^b^	—	78 (31.8)
Using traffic lights/experiential task page	—	—	101 (41.2)
FLICC task and aims	—	—	122 (49.8)
Set their own goal	—	—	89 (36.3)

^a^FLICC: Front-of-pack food Labels: Impact on Consumer Choice.

^b^Not applicable.

## Discussion

### Principal Findings

The FLICC pilot trial was an example of a partnership between academia and the supermarket industry to allow for a randomized trial of a behavior change intervention that utilized supermarket loyalty card data for(1) recruitment, (2) provision of tailored feedback on previous purchases, and (3) objective and remote measurement of participant food purchases. The pilot study showed that remote delivery of dietary studies across wide populations allows for speedy recruitment (albeit recruiting only 1% of the participant pool), that measurement of outcomes using loyalty cards can lead to very high retention rates, but that engagement with remotely delivered digital behavior change interventions can be low. The trial did not provide evidence to suggest this specific intervention would be effective at changing purchasing behavior, but the process of conducting the trial has revealed much information about using supermarket loyalty card data for both the delivery of public health interventions and for trials of their effectiveness—issues that are growing in relevance [31-33].

Supermarket loyalty card data provide benefits to public health research; however, the use of such data is rare in the evaluation of public health interventions [8-11]. This study revealed both benefits and limitations attached to the use of loyalty card data. The benefits include high retention rates and the potential to recruit quickly from a wide population. Limitations include generic problems with drawing conclusions about consumption from purchase data (discussed below) and specific issues associated with data sharing between academia and the supermarket industry. Loyalty card data are commercially sensitive, and supermarkets have a duty to ensure that individual-level data are handled with an appropriate level of security as consumers have privacy concerns over the handling of loyalty card data [7]. Recruiting from a loyalty card database means we had little control over recruitment methods, and a low participation rate may not have resulted in a representative sample. These issues are discussed here to highlight practical issues that researchers will need to consider if pursuing similar arrangements in the future. In the case of the FLICC study, the agreement of terms and conditions for transferal of data and the subsequent signing of contracts took over 12 months, and this was far longer than anticipated by either party. This caused significant amendments to the planned timetable for the study. For practical reasons, the participating supermarket was only able to provide us with 2 data extracts over the course of the project, which precluded us from providing participants with multiple updates on their shopping history as the trial progressed. For such collaborative studies to achieve the most success in the future, we advise that at an early stage, all collaborative partners develop an understanding of what can and cannot be delivered by the collaboration. We are grateful that the participating supermarket remained flexible to our needs throughout the research process, without which this study would not have been possible.

The main strengths of our study are the strong internal validity associated with the RCT design, the remote nature of the delivery of the intervention and collection of data (allowing for scalability of an intervention if shown to be effective), and the use of the supermarket loyalty card dataset for recruitment, which allowed quick and efficient recruitment of a large number of participants. The pilot study was able to answer questions about the practicality and feasibility of conducting trials in a supermarket setting in partnership with a supermarket chain, highlighting both the advantages and disadvantages of such a partnership while providing evidence on essential study features that could not have been known in advance. Of the examples, 1 includes the average amount of food purchasing that was recorded by study participants. In the FLICC study, the average amount of money spent on food captured by the loyalty cards was less than £20 per week, whereas the average amount of money spent on food and nonalcoholic drinks in the United Kingdom is £56.80 per household per week [2], which suggests that purchases in our study were a subset of all food purchases made by the participants. This has implications for whether our outcome measures truly reflect consumption behavior. Supermarket loyalty cards can only measure food purchases when a loyalty card is used, which may not be at every occasion that participants visit the supermarket. They also restrict the range of a study to include purchases in 1 supermarket chain only, which may not reflect shopping behavior in many people who regularly frequent more than 1 supermarket chain. They may also be shared by friends and family, so may not collect data from the individual recruited to the study [34]. Although only own-brand ready meal and pizza purchases were included for our analyses and some other branded products may have traffic light labels, we have shown that 79.3% of all purchases were of own-brand products, and thus, we have captured the vast majority of relevant purchasing behavior.

Another limitation was the amount of MOD for the primary outcome variable, which was a result of participants’ loyalty card data showing zero purchases of own-brand ready meals or pizzas in T1 and/or T2 (despite participants’ self-reporting at recruitment that they were frequent purchasers of these products). Ideally, we would have filtered the recruitment email so that only individuals who have shown frequent purchases of own-brand ready meal and pizzas in their loyalty card data were contacted to take part in the study—unfortunately this was not possible, as the recruitment email was delivered by a market research company aligned with the participating supermarket who had access to geographic and demographic data on loyalty card holders but not on previous purchases. Our screening question at recruitment was “Thinking about the last 6 months, on average have you purchased either Ready Meals or Pizzas at least twice per month? (It doesn’t matter if the Ready Meals or Pizzas were for you or for other members of your household).” However, 22% of the recruited participants did not have any records of own-brand ready meal or pizza purchases on their loyalty card data from the previous 6 months (T-1). This was a far higher percentage than we anticipated and increased to 52% during the intervention period (T1). Our predetermined statistical analysis plan stated that we would conduct analyses with imputation for the primary outcome variable but because of the amount of MOD, the imputation effectively overwhelms the analysis.

We measured engagement with our digital intervention using Web analytic tools. Similar methods are now regularly used to measure the engagement of participants with Web-based interventions [35]. We found that overall engagement with the intervention was low but at a similar level (50%) found in a systematic review of use of Web-based behavior change interventions [36]. Other studies have found that engagement with digital interventions can be boosted by telephone-based coaching [37] or professional support [38]; however, this would affect the potential reach of such an intervention delivered at scale. Provision of frequent updates of the intervention, which was not possible in our study because of the data-sharing relationship with the participating supermarket, has also been shown to increase engagement with digital behavior change interventions [38,39].

Other trials have investigated the impact of remotely delivered tailored feedback on dietary behavior and found more encouraging results. Alexander et al [40] conducted an RCT of 2540 participants, where arms 2 and 3 received a website with tailored feedback on previous diet compared with a control of a nontailored dietary advice website, and found that the intervention increased consumption of fruit and vegetables reported in a food frequency questionnaire by 2.7 and 2.8 portions per day, respectively, compared with 2.3 portions per day for the control group (*P*=.177 for arm 2, *P*=.050 for arm 3). Huang et al [41] found that a website that provided tailored advice resulted in a reduction in saturated fat of 0.66% of food energy in food purchases from an online supermarket compared with a control of nontailored advice (*P*<.001), in a randomized trial of 497 participants. Tapper et al [42] found an increase in fruit and vegetable consumption reported in a food frequency questionnaire in intervention compared with control group (*P*=.08) in an RCT of a website providing tailored dietary feedback with 100 participants. The interventions studied in these trials were more intensive and required a larger time commitment from participants than the intervention in the FLICC study. A recent randomized trial of a phone app that provides feedback on potential food purchases (suggesting switches for lower salt products) found a significant reduction of salt in food purchases of 0.3 g/MJ (95% CI 0.03-0.58) in a study of 66 adults with diagnosed cardiovascular disease [43], suggesting feedback on nutritional content of foods combined with proposed alternatives could be an effective mechanism for improving diets.

### Conclusions

Although the FLICC study did not find evidence of an impact of the intervention on food purchasing behavior, the unique methods used in this pilot trial are informative for future studies that plan to use supermarket loyalty card data in collaboration with supermarket partners. The experience of the trial showcases the possibilities and challenges associated with the use of loyalty card data in public health research.

## Appendix

Multimedia Appendix 1 Study participant flowchart and results of prespecified subanalysis by socioeconomic status.

Multimedia Appendix 2 CONSORT 2010 checklist for the FLICC study.

## Abbreviations

FLICC: Front-of-pack food Labels: Impact on Consumer Choice
FOP: front of pack
MOD: missing outcome data
NCD: noncommunicable disease
NS-SEC: National Statistics Socio-economic Classification
RCT: randomized controlled trial
