# Supplementary material for: A Pilot Randomized Controlled Trial of a Digital Intervention Aimed at Improving Food Purchasing Behavior: The Front-of-Pack Food Labels Impact on Consumer Choice Study
Source: JMIR Form Res. 2019 Apr 8;3(2):e9910. doi: 10.2196/formative.9910 (PMC6482590; doi:10.2196/formative.9910)
Supplement: Multimedia Appendix 1 [file formative_v3i2e9910_app1.pdf]

Supplementary Material

Figure S1. FLICC trial flowchart, indicating the number of participants (or potential participants) engaged at each stage of the trial

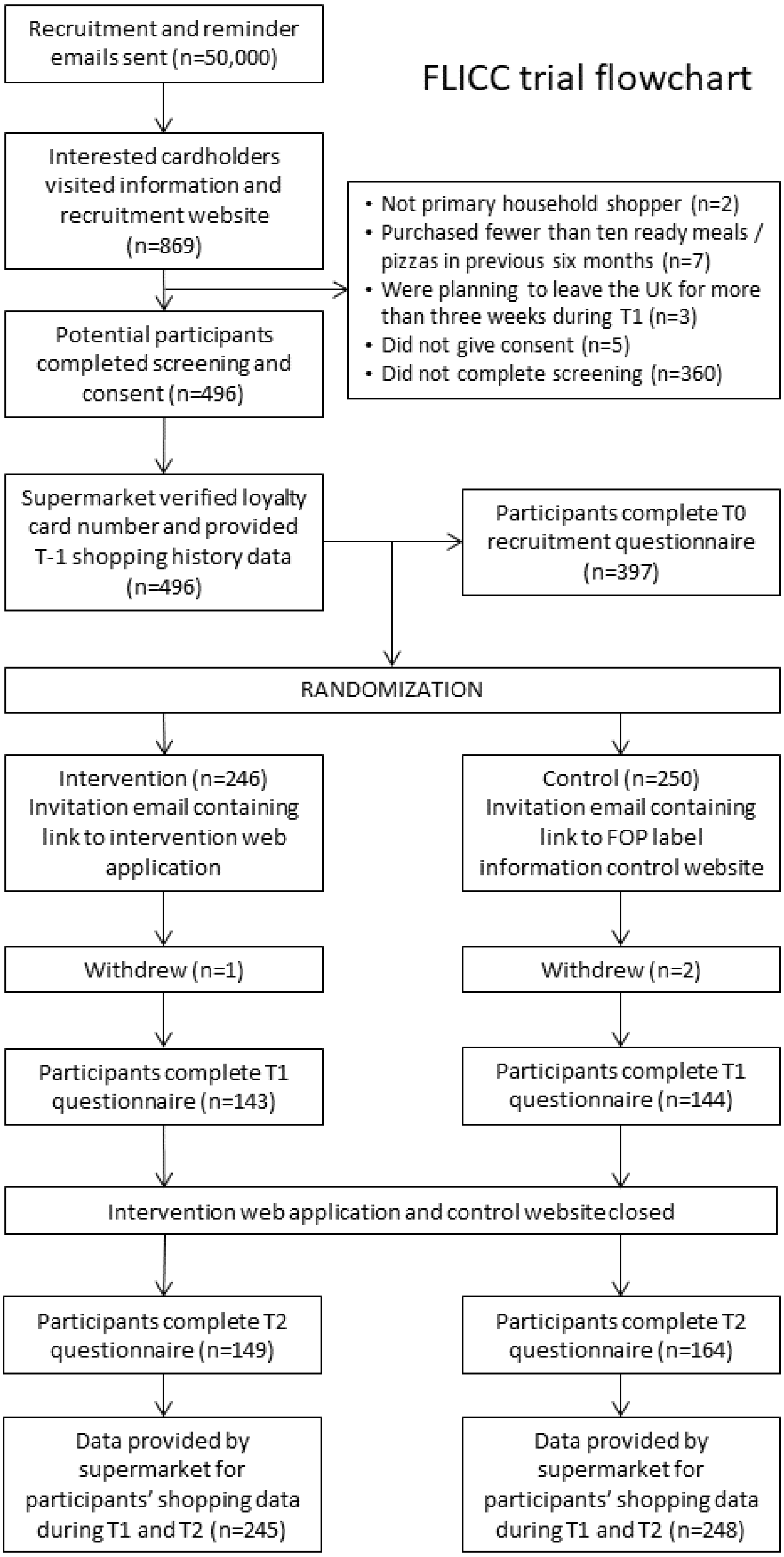

Table S2. Primary outcome measure results – healthiness of ready meals and pizzas purchased by intervention and control arms in three study phases, sub-analyses by socioeconomic group.[a]

	**T-1 Mean (SE)**	**T1 Mean (SE)**	**T2 Mean (SE)**
**Low SES (n = 92)**			
Control	0.568 (0.019)	0.561 (0.024)	0.539 (0.025)
Intervention	0.577 (0.019)	0.587 (0.028)	0.569 (0.027)
p[b]	0.658	0.376	0.397
**High SES (n = 300)**			
Control	0.575 (0.011)	0.573 (0.012)	0.561 (0.015)
Intervention	0.565 (0.011)	0.574 (0.013)	0.552 (0.013)
p[b]	0.523	0.597	0.599

[a] NB: Healthiness score runs from 0 to 1, with higher score indicating healthier food purchases. Low SES = groups 3, 4 and 5 of NS-SEC; High SES = groups 1 and 2 of NS-SEC.

[b]Results of ANCOVA comparing intervention and control adjusted for sex, dependent children and healthiness of ready meals and pizzas purchased at T-1.
